# Trajectories of glycaemia, insulin sensitivity and insulin secretion in South Asian and white individuals before diagnosis of type 2 diabetes: a longitudinal analysis from the Whitehall II cohort study

**DOI:** 10.1007/s00125-017-4275-6

**Published:** 2017-04-13

**Authors:** Adam Hulman, Rebecca K. Simmons, Eric J. Brunner, Daniel R. Witte, Kristine Færch, Dorte Vistisen, Satoyo Ikehara, Mika Kivimaki, Adam G. Tabák

**Affiliations:** 1 Department of Public Health, Aarhus University, Building 1260, Bartholins Allé 2, 8000 Aarhus C, Denmark; 2 Danish Diabetes Academy, Odense, Denmark; 3 Department of Medical Physics and Informatics, University of Szeged, Szeged, Hungary; 4 MRC Epidemiology Unit, University of Cambridge School of Clinical Medicine, Box 285, Institute of Metabolic Science, Cambridge Biomedical Campus, Cambridge, CB2 0QQ UK; 5 Aarhus Institute of Advanced Studies, Aarhus University, Aarhus, Denmark; 6 Department of Epidemiology and Public Health, University College London, London, UK; 7 Steno Diabetes Center Copenhagen, Gentofte, Denmark; 8 Department of Hygiene and Public Health, Osaka Medical College, Osaka, Japan; 9 First Department of Medicine, Faculty of Medicine, Semmelweis University, Budapest, Hungary

**Keywords:** Cohort study, Ethnicity, Glucose, Glycaemic trajectory, Insulin, South Asia

## Abstract

**Aims/hypothesis:**

South Asian individuals have reduced insulin sensitivity and increased risk of type 2 diabetes compared with white individuals. Temporal changes in glycaemic traits during middle age suggest that impaired insulin secretion is a particular feature of diabetes development among South Asians. We therefore aimed to examine ethnic differences in early changes in glucose metabolism prior to incident type 2 diabetes.

**Methods:**

In a prospective British occupational cohort, subject to 5 yearly clinical examinations, we examined ethnic differences in trajectories of fasting plasma glucose (FPG), 2 h post-load plasma glucose (2hPG), fasting serum insulin (FSI), 2 h post-load serum insulin (2hSI), HOMA of insulin sensitivity (HOMA2-S) and secretion (HOMA2-B), and the Gutt insulin sensitivity index (ISI_0,120_) among 120 South Asian and 867 white participants who developed diabetes during follow-up (1991–2013). We fitted cubic mixed-effects models to longitudinal data with adjustment for a wide range of covariates.

**Results:**

Compared with white individuals, South Asians had a faster increase in FPG before diagnosis (slope difference 0.22 mmol/l per decade; 95% CI 0.02, 0.42; *p* = 0.03) and a higher FPG level at diagnosis (0.27 mmol/l; 95% CI 0.06, 0.48; *p* = 0.01). They also had higher FSI and 2hSI levels before and at diabetes diagnosis. South Asians had a faster decline and lower HOMA2-S (log_*e*_-transformed) at diagnosis compared with white individuals (0.33; 95% CI 0.21, 0.46; *p* < 0.001). HOMA2-B increased in both ethnic groups until 7 years before diagnosis and then declined; the initial increase was faster in white individuals. ISI_0,120_ declined steeply in both groups before diagnosis; levels were lower among South Asians before and at diagnosis. There were no ethnic differences in 2hPG trajectories.

**Conclusions/interpretation:**

We observed different trajectories of plasma glucose, insulin sensitivity and secretion prior to diabetes diagnosis in South Asian and white individuals. This might be due to ethnic differences in the natural history of diabetes. South Asian individuals experienced a more rapid decrease in insulin sensitivity and faster increases in FPG compared with white individuals. These findings suggest more marked disturbance in beta cell compensation prior to diabetes diagnosis in South Asian individuals.

## Introduction

Pathophysiological changes leading to type 2 diabetes and macrovascular complications begin to develop well before current cut-off levels for impaired fasting glucose, impaired glucose tolerance and diabetes [1–3]. This increased risk differs by ethnic group. In the UK, individuals of South Asian descent, for example, have a substantially higher risk of developing type 2 diabetes compared with individuals of European descent [4]. There is robust evidence that reduced insulin sensitivity contributes to this elevated risk [5–7]. Beta cell dysfunction is also a plausible aetiological factor explaining elevated risk among South Asian individuals [8, 9]. First, fetal undernutrition is common in Asian populations, which could lead to reduced pancreatic growth and lower insulin secretion levels [3, 10]. Second, reduced insulin sensitivity is observed in South Asians from early life and could contribute to inadequate insulin secretion through gluco- and lipotoxicity [11]. However, conducting population-based studies on beta cell function is challenging because measures of insulin secretion based on fasting plasma glucose (FPG) or the OGTT do not account for the dependence of insulin secretion on blood glucose levels and underlying insulin sensitivity [12, 13].

Recent advancements in longitudinal modelling allow estimation of changes in beta cell function by examining trajectories of insulin sensitivity and insulin secretion together over time. We have previously identified a long ‘compensatory’ period in the pathophysiology leading to diabetes. This is characterised by increasing insulin secretion, which compensates for insulin resistance while keeping glucose values stable [14]. We have also identified subgroups with different trajectories of insulin sensitivity and secretion prior to the development of diabetes, suggesting important heterogeneity in the relative contributions of insulin sensitivity and secretion before diagnosis of type 2 diabetes [15]. Longitudinal modelling of age-related changes in insulin sensitivity and insulin secretion among individuals without diabetes has shown a lack of compensatory increase in insulin secretion among South Asian compared with white individuals, supporting the hypothesis of a reduced pancreatic functional reserve in this high-risk group [3]. However, ethnic differences in the natural history of early changes in glucose metabolism prior to incident type 2 diabetes are not well understood, precluding optimal timing for screening and prevention activities among South Asian people.

In this prospective study with repeat assessments of glycaemic traits, we used longitudinal modelling to examine ethnic differences in trajectories of FPG and 2 h post-load plasma glucose (2hPG), fasting serum insulin (FSI) and 2 h post-load insulin (2hSI), HOMA of insulin sensitivity (HOMA2-S) and secretion (HOMA2-B), and the Gutt insulin sensitivity index (ISI_0,120_) prior to diabetes diagnosis in a UK population of Europid or South Asian ethnicity.

## Methods

### Study population and design

The Whitehall II study was set up in 1985. All non-industrial British civil servants aged 35–55 years and working in London offices were invited to take part. The response rate was 73%, with 10,308 participants attending an initial clinical examination and completing self-administered questionnaires (phase 1). During follow-up, 5 yearly clinical examinations were performed and additional questionnaire-only phases were conducted until 2012–2013 (phase 11). The University College London Ethics Committee reviewed and approved the study. Written informed consent was obtained from all participants at each study phase.

OGTTs were first performed at phase 3 (1991–1994), serving as the baseline for our analyses (*n* = 8815). OGTTs took place in either the morning or afternoon, following ≥8 h and ≥5 h of fasting, respectively. We excluded the following individuals: those from ethnic groups other than South Asian or white (*n* = 397); those with prevalent diabetes or missing follow-up data on diabetes status (*n* = 516); those who remained free of diabetes during the study (*n* = 6801); those who did not provide any fasting samples according to the Whitehall II protocol (*n* = 47); and those with missing covariate information, e.g. data on BMI, diet, physical activity and employment grade (*n* = 67). The final sample consisted of 120 South Asian and 867 white individuals who developed diabetes during a median (interquartile range) follow-up of 13.2 (9.9–16.4) years. Diabetes was diagnosed either by a doctor outside the study or during the clinical examination according to WHO epidemiological criteria [16, 17]. FPG values were available in all phases of the study; 2hPG was available in all but the final phase (phase 11). HbA_1c_ measures were available from phase 7 onwards. For doctor-diagnosed diabetes, the date of diagnosis was defined as the midpoint between the date of the last screening and the date of the phase when the participant reported diagnosed diabetes.

### Measurements

Fasting and 2 h post-load venous blood samples were taken during a 75 g OGTT according to standardised protocols. Blood glucose was measured with the glucose oxidase method (YSI Corporation, Yellow Springs, OH, USA). Serum insulin was measured with an in-house human insulin RIA and later with an ELISA kit (Dako Cytomation, Ely, UK) [14]. HOMA2-S and HOMA2-B were calculated with the HOMA2 calculator, version 2.2 (www.dtu.ox.ac.uk/homacalculator/index.php) using FPG and FSI values [18]. ISI_0,120_ was calculated using the equation developed by Gutt et al [19]. BMI was measured according to standardised protocols. Ethnicity was defined using Office for National Statistics 1991 census types. We used self-reported ethnicity from phase 5; missing data were complemented by observer-assigned ethnicity from phase 1. Rates of agreement between observer-assigned and self-reported ethnicity were high (93% for South Asian and 99.3% for white individuals). South Asians were defined as individuals of Indian, Sri Lankan, Pakistani or Bangladeshi ethnic origin. We used British Civil Service employment grade as a measure of occupational status. This was grouped into three categories: high (senior administrators), intermediate (executives, professionals and technical staff) and low (clerical and office support staff). Physical activity was assessed by questions on the frequency and duration of participation in physical activity at different intensities and grouped into three categories: inactive, moderately active and active. Global dietary patterns were derived from the type of bread and milk most frequently consumed and the frequency of fruit and vegetable consumption (on an eight-point scale). The dietary score classified participants into three groups: healthy (three points), moderately healthy (between four and seven points) or unhealthy (eight or more points). Assessment of lifestyle factors has been described previously [20].

### Statistical analyses

We described and compared baseline characteristics between the two ethnic groups. FSI, 2hSI, HOMA2-S, HOMA2-B and ISI_0,120_ were log_*e*_-transformed because of their skewed distribution. Linear (*t*), quadratic (*t* ^2^) or cubic (*t* ^3^) trajectories were assessed by fitting mixed-effects models, depending on which form best fitted the data. These models use all available data and do not require the same number of measurements per person or the same time points. They also take into account the within-person correlation arising from the longitudinal nature of the data. The time variable of the models was defined as follows: the date of diagnosis was set to year 0 and all measurements that were taken before this date were included retrospectively until the first clinical examination. First, we modelled differences in glycaemic trajectories between white and South Asian individuals by including ethnicity as a binary variable (South Asian/white) in the models along with its interactions with all the time terms (linear, quadratic, cubic). All models were adjusted for age at diagnosis and sex. When the ethnicity by cubic time interaction was not significant, the model was refitted without this term. Then we tested the ethnicity by quadratic time term the same way and decided on inclusion based on statistical significance of the interaction term. The ethnicity by first order time interaction was always retained in the model regardless of its statistical significance. Second, we further adjusted the models for time-varying BMI, diet and physical activity, and for baseline employment grade. To compare the models, we used the same specification for these multivariable adjusted models as for the age- and sex-adjusted models. In a sensitivity analysis, we compared all models with and without adjustment for age and BMI. All statistical analyses were performed in R, version 3.0.1 (R Foundation for Statistical Computing, Vienna, Austria). Statistical significance was inferred at a two-tailed *p* value <0.05.

## Results

The 120 South Asian and 867 white individuals who developed diabetes between 1991 and 2013 contributed a total of 2140 fasting measurements to our analysis. Median follow-up time was 13.1 years for South Asian and 13.2 years for white individuals. The median age at diabetes diagnosis was 63 years; this did not differ by ethnic group (*p* = 0.97). There was a small difference in the mode by which South Asian and white individuals were diagnosed, i.e. by screening or clinical diagnosis (screening 43% vs 54%, respectively; *p* = 0.03). There were no ethnic differences in the proportion of individuals diagnosed via OGTT in the morning or afternoon (*p* = 0.73).

Participant characteristics at baseline are summarised by ethnic group in Table 1. Compared with their counterparts of European descent, South Asian participants were 1 year older and more likely to be female. They were more likely to have a low occupational grade, exhibit low physical activity levels and follow an unhealthy diet.

**Table 1:** Baseline characteristics of study participants by ethnicity

Variable	White	South Asian
*n*	867	120
Age, years	50 (45–56)	51 (46–55)
Male, %	74	63
BMI, kg/m^2^	26.3 (24.4–29.3)	24.9 (23.5–28.0)
FPG, mmol/l	5.4 (5.1–5.8)	5.3 (5.1–5.7)
2hPG, mmol/l	6.1 (5.0–7.7)	6.2 (5.3–7.6)
FSI, pmol/l	7.4 (5.0–11.8)	10.0 (5.8–14.9)
2hSI, pmol/l	54.5 (31.5–87.9)	83.0 (58.9–114.4)
HOMA2-B	82.0 (65.0–109.8)	98.6 (70.7–127.1)
HOMA2-S	96.9 (62.8–142.5)	75.6 (50.8–126.6)
ISI_0,120_	70.8 (53.8–92.7)	62.6 (51.2–75.5)
Occupational grade, %		
High	38	7
Intermediate	47	57
Low	15	36
Physical activity, %		
Active	49	34
Moderately active	31	30
Inactive	19	35
Dietary pattern, %		
Healthy	14	13
Moderately healthy	79	67
Unhealthy	7	20

Values are per cent or median (Q1–Q3)

**Fig. 1:**
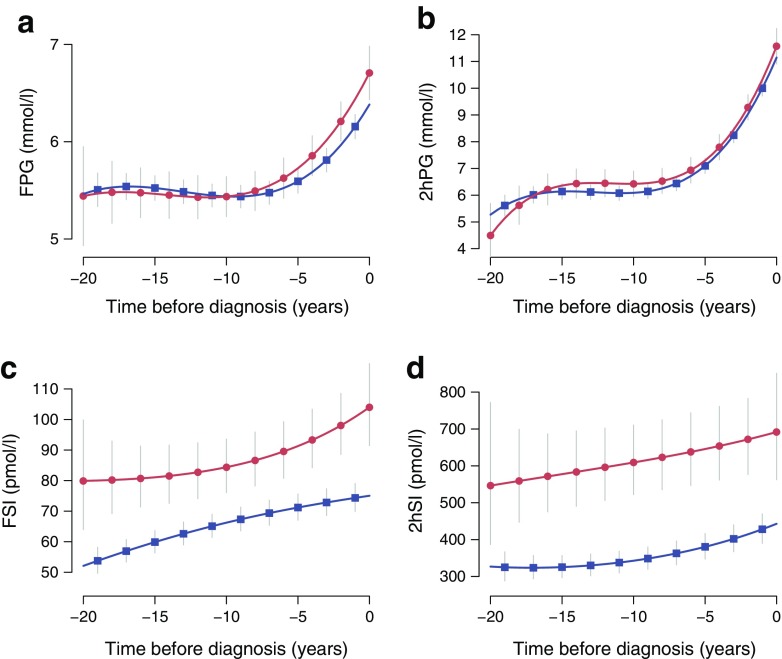
Trajectories of (**a**) FPG, (**b**) 2hPG, (**c**) FSI and (**d**) 2hSI, with 95% CIs, by ethnic group (red circles, South Asians; blue squares, whites). All models were adjusted for age, sex, BMI, employment grade, diet and physical activity. Insulin values were log_*e*_-transformed before fitting the models and then transformed back to the original scale

**Fig. 2:**
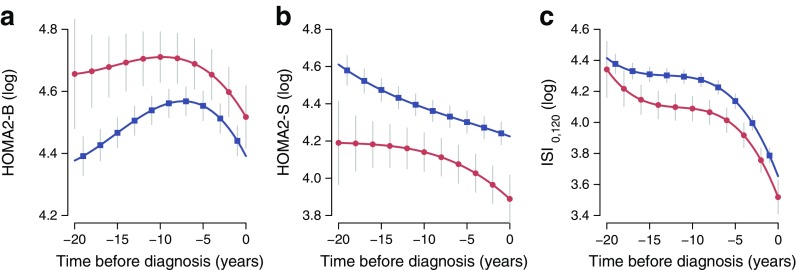
Log_*e*_-transformed (**a**) HOMA2-B, (**b**) HOMA2-S and (**c**) ISI_0,120_ trajectories with 95% CIs, by ethnic group (red circles, South Asians; blue squares, whites). All models were adjusted for age, sex, BMI, employment grade, diet and physical activity

Figure 1 shows glucose and insulin trajectories (FPG, 2hPG, FSI and 2hSI) prior to diagnosis of diabetes in each ethnic group, while Fig. 2 shows the insulin sensitivity and secretion trajectories (HOMA2-S, HOMA2-B and ISI_0,120_). Coefficients of the best fitting trajectories are shown in Tables 2 and 3. Cubic models provided better model fit for FPG, 2hPG, HOMA2-B and ISI_0,120_. By contrast, adding a cubic term did not improve model fit for FSI and HOMA2-S. A linear model best described changes in 2hSI over time.

**Table 2:** Mixed-effects models of change for FPG, 2hPG, FSI and 2hSI by ethnic group (comparing South Asian and white individuals)

	FPG	2hPG	FSI (log_*e*_)	2hSI (log_*e*_)
Model 1				
Intercept	6.44 (6.34, 6.53)***	11.31 (11.08, 11.54)***	4.36 (4.31, 4.41)***	6.08 (6.02, 6.14)***
SA	0.21 (0.00, 0.42)*	0.24 (−0.26, 0.74)	0.21 (0.07, 0.35)**	0.39 (0.22, 0.56)***
*t*	0.25 (0.21, 0.29)***	1.26 (1.17, 1.35)***	0.01 (0.00, 0.02)*	0.02 (0.02, 0.03)***
*t* × SA	0.022 (0.002, 0.042)*	0.022 (−0.024, 0.068)	0.029 (−0.001, 0.060)	−0.005 (−0.020, 0.009)
*t* ^2^	0.020 (0.015, 0.026)***	0.102 (0.090, 0.114)***	−0.0009 (−0.0015, −0.0004)**	–
*t* ^2^ × SA	–	–	0.0018 (0.0002, 0.0035)*	–
*t* ^3^	0.0005 (0.0003, 0.0007)***	0.0027 (0.0023, 0.032)***	–	–
*t* ^3^ × SA	–	–	–	–
Model 2				
Intercept	6.39 (6.25, 6.52)***	11.15 (10.83, 11.46)***	4.32 (4.25, 4.38)***	6.05 (5.96, 6.15)***
SA	0.27 (0.06, 0.48)*	0.39 (−0.11, 0.90)	0.33 (0.20, 0.45)***	0.48 (0.32, 0.64)***
*t*	0.25 (0.21, 0.29)***	1.26 (1.17, 1.35)***	0.01 (−0.00, 0.02)	0.02 (0.01, 0.02)***
*t* × SA	0.022 (0.002, 0.042)*	0.021 (−0.025, 0.067)	0.022 (−0.005, 0.049)	−0.006 (−0.020, 0.007)
*t* ^2^	0.021 (0.015, 0.026)***	0.103 (0.091, 0.115)***	−0.0006 (−0.0011, −0.0000)*	–
*t* ^2^ × SA	–	–	0.0014 (−0.0001, 0.0029)	–
*t* ^3^	0.0005 (0.0003, 0.0007)***	0.0027 (0.0023, 0.0032)***		–
*t* ^3^ × SA	–	–		–

Values are model coefficients (95% CI)

Model 1: age (60 years), sex (male); model 2: age (60 years), sex (male), BMI (28 kg/m^2^), employment grade (intermediate), diet (moderately healthy), physical activity (moderately active)

Insulin values were log_*e*_-transformed before analysis due to the skewed distribution

*p < 0.05, **p < 0.01, ***p < 0.001

SA, South Asian; *t*, time

**Table 3:** Mixed-effects models of change for HOMA2-B, HOMA2-S and ISI_0,120_ by ethnic group (comparing South Asian and white individuals)

	HOMA2-B (log_*e*_)	HOMA2-S (log_*e*_)	ISI_0,120_ (log_*e*_)
Model 1			
Intercept	4.39 (4.36, 4.44)***	4.18 (4.13, 4.24)***	3.63 (3.59, 3.67)***
SA	0.03 (−0.06, 0.12)	−0.22 (−0.36, −0.08)**	−0.10 (−0.19, −0.02)*
*t*	−0.05 (−0.07, −0.04)***	−0.016 (−0.026, −0.006)**	−0.14 (−0.16, −0.13)***
*t* × SA	−0.007 (−0.014, −0.000)*	−0.032 (−0.062, −0.001)*	0.001 (−0.007, 0.008)
*t* ^2^	−0.005 (−0.007, −0.003)***	0.0007 (0.0002, 0.0013)*	−0.011 (−0.013, −0.009)***
*t* ^2^ × SA	–	−0.0019 (−0.0036, −0.0002)*	–
*t* ^3^	−0.00011 (−0.00017, −0.00004)**	–	−0.0003 (−0.0003, −0.0002)***
*t* ^3^ × SA	–	–	–
Model 2			
Intercept	4.39 (4.34, 4.45)***	4.23 (4.16, 4.29)***	3.66 (3.60, 3.71)***
SA	0.10 (0.02, 0.18)*	−0.33 (−0.46, −0.21)***	−0.16 (−0.24, −0.07)***
*t*	−0.052 (−0.066, −0.039)***	−0.012 (−0.021, −0.002)*	−0.14 (−0.16, −0.13)***
*t* × SA	−0.007 (−0.014, −0.001)*	−0.024 (−0.051, 0.003)	0.001 (−0.006, 0.009)
*t* ^2^	−0.005 (−0.006, −0.003)***	0.0003 (−0.0002, 0.0009)	−0.011 (−0.013, −0.009)***
*t* ^2^ × SA	–	−0.0014 (−0.0029, 0.0001)	–
*t* ^3^	−0.00010 (−0.00016, −0.00004)**	–	−0.0003 (−0.0004, −0.0002)***
*t* ^3^ × SA	–	–	–

Values are model coefficients (95% CI)

Model 1: age (60 years), sex (male); model 2: age (60 years), sex (male), BMI (28 kg/m^2^), employment grade (intermediate), diet (moderately healthy), physical activity (moderately active)

All outcome variables were log_*e*_-transformed before analysis due to the skewed distribution

*p < 0.05, **p < 0.01, ***p < 0.001

SA, South Asian; *t*, time

Among both ethnic groups, there were small increases in FPG over time, with a slightly larger increase among South Asian compared with white individuals (slope difference 0.22 mmol/l per decade; 95% CI 0.02, 0.42; *p* = 0.03) (Table 2). This group exhibited higher FPG levels at diagnosis (0.27 mmol/l; 95% CI 0.06, 0.48; *p* = 0.01). Most of the increases in FPG were observed during the 3–4 years before diabetes diagnosis.

There were large increases in 2hPG over time in both ethnic groups. Trajectories of 2hPG did not differ between ethnic groups (slope difference 0.21 mmol/l per decade; 95% CI −0.25, 0.67; *p* = 0.37) (Table 2). The 2hPG cubic trajectory was characterised by a steep rise from approximately 7 to 12 mmol/l in the last 6 years prior to diagnosis. Levels of FSI and 2hSI were higher among South Asian compared with white individuals before and at diabetes diagnosis. The shape of the 2hSI trajectory was similar in both ethnic groups, while the FSI curve exhibited some differences (*p* < 0.05 for *t* ^2^ × ethnicity interaction).

HOMA2-S levels were higher among white compared with South Asian individuals throughout the follow-up period (geometric mean difference 28%) (Table 3). There was a linear decrease in HOMA2-S during follow-up in both ethnic groups, with levels declining from 79.8 to 74.2 among white individuals and from 63.0 to 53.1 among South Asians 10 years before diagnosis. This decline was steeper in the South Asian group from 10 years before diagnosis; however, ethnic difference in the shape of the trajectories became non-significant in the most adjusted model.

Insulin secretion levels were higher among South Asian compared with white individuals throughout the follow-up period. There was a modest increase in HOMA2-B levels in both ethnic groups until 7 years before diagnosis. This initial increase was 7.3% (95% CI 0.6, 14.1) faster among white individuals. Insulin secretion then declined in both ethnic groups in a quadratic trajectory until diagnosis (Table 3).

ISI_0,120_ levels were 17.4% (95% CI 7.3, 31.0; geometric mean difference) lower among South Asian compared with white individuals before and at diagnosis; however, the shape of the cubic trajectories was similar between the two ethnic groups (Table 3).

In a sensitivity analysis comparing all models with and without adjustment for age and BMI, the shapes of the trajectories were very similar; only the intercept differences were altered (data not shown).

## Discussion

Repeat data from UK adults of European and South Asian ethnic origin showed changes in glucose levels, insulin sensitivity and insulin secretion several years before diabetes diagnosis in both ethnic groups. FPG was found to increase faster and reach higher values in South Asian compared with white individuals. Insulin sensitivity levels were lower throughout follow-up and fell more rapidly among South Asian than among white individuals. A compensatory increase in insulin secretion alongside decreasing insulin sensitivity was observed among white individuals up to 7 years before diagnosis. However, this trend was not seen in South Asians, who already had higher insulin secretion levels 20 years before they were diagnosed with diabetes. This suggests that they managed to compensate for their higher insulin resistance for many years until a steep decline in insulin secretion (and probable beta cell exhaustion) in the 5–7 years before diagnosis. In combination, these findings support the hypothesis that South Asians exhibit long-term beta cell compensation for chronic insulin resistance from childhood and that they are unable to produce further beta cell compensation in response to decreasing insulin sensitivity above 60 years of age [3]. Whether these differences are innate or related to the deleterious effects of some environmental factors is a question that our study is currently unable to answer.

Our data support previous studies showing that the increase in FPG before a diabetes diagnosis is rapid and occurs mostly in the 3–4 years before diagnosis [14, 21–23], and when beta cell dysfunction is present [15, 24, 25]. A novel finding of our analysis is the steeper increasing FPG trajectory in South Asians, suggesting a shorter window of opportunity for prevention at any given FPG value. As clinical screenings were performed at regular intervals in both South Asian and white individuals, the lower rate of screen-detected diabetes among South Asians is also compatible with the steeper increase in FPG we observed. Given the potential for underdiagnosis among South Asians, our graphs may therefore underestimate the real slope differences. This supports our conjecture that South Asians appear to have an accelerated development of type 2 diabetes compared with white individuals.

In both ethnic groups, 2hPG followed a cubic increase over time, with an approximate 5 mmol/l increase in the last 8 years before diagnosis. Again, our data support previous smaller scale studies showing a rapid rise in 2hPG and a similar slope before diagnosis [14, 21, 26]. The observed ethnic difference in the 2hPG slope was of the same magnitude as the FPG slope. However, this translated to a much smaller, and in the most adjusted model non-significant, relative difference between ethnicities. Whether this finding relates to lifestyle or obesity or is simply a power issue requires further study.

Our results confirm the essential role of low insulin sensitivity in the development of incident diabetes [14]. We observed a decline in insulin sensitivity in both ethnic groups as the cohort aged and a steep decrease in the 7 years preceding diagnosis among South Asians. South Asian individuals had lower insulin sensitivity than white individuals up to 20 years before they were diagnosed with diabetes. This finding supports previous literature showing decreased insulin sensitivity from a young age among South Asians; this decrease appears decades before the manifestation of diabetes [3, 7, 27, 28]. Plausible explanations for the early emergence of decreased insulin sensitivity include abdominal fat distribution and low muscle mass, unhealthy lifestyles, low levels of adiponectin and high levels of proinflammatory cytokines. Our multivariable analyses were adjusted for obesity (BMI), diet and physical activity, yet the differences between the South Asian and white participants remained for FPG and measures of insulin sensitivity and secretion.

We observed higher levels of HOMA2-B among South Asians compared with white individuals at baseline, probably reflecting compensation for their lower insulin resistance. This group had relatively stable FPG levels suggesting good compensation for many years until a rapid decline in insulin secretion in the 5–7 years before diagnosis, contributing to the more rapidly increasing FPG trajectory. This trend may be explained by a number of hypotheses, including fetal undernutrition among Asian populations leading to reduced beta cell reserve (though perhaps only among first-generation South Asians in our study sample) [10], long-term beta cell compensation for chronic insulin resistance from childhood [9], and/or increasing FPG and 2hPG levels producing glucose toxicity and consequent beta cell failure [12, 13].

### Strengths and limitations

We used repeat measures from a large, occupational British cohort to compare ethnic differences in trajectories of glycaemic measures prior to diagnosis of type 2 diabetes. Data were collected over 23 years using robust standard operating procedures. This long follow-up time allowed us to examine long-term changes in a very well phenotyped cohort. There was a change in the diagnostic criteria for diabetes in the last period of the study (i.e. the introduction of HbA_1c_). However, this is only expected to account for a few cases of clinically diagnosed diabetes and is unlikely to be differential by ethnic group. Relatedly, while diabetes diagnosis was not confirmed by a repeat OGTT, and we used the midpoint between study visits as the date of (clinical) diagnosis of diabetes, we expect this small risk of misclassification to be similar between the two ethnic groups.

Our analysis approach allowed us to examine non-linear patterns of change while taking into account the within-person correlation arising from the longitudinal structure of the data. Since gold standard measures of insulin sensitivity are not feasible to use in large epidemiological studies, we used the well-accepted and extensively validated HOMA insulin sensitivity measure and the ISI_0,120_ index [18, 19]. Our measure of HOMA insulin secretion is less widely used [18]. The HOMA models mostly reflect hepatic insulin resistance and steady-state insulin secretion as they use fasting values for estimation. By contrast, ISI_0-120_, which includes both fasting and 2 h glucose and insulin concentrations, might be a better reflection of whole-body insulin sensitivity. While hepatic insulin resistance is strongly associated with fat and muscle insulin resistance, elevated insulin secretion in the fasting state is a late marker of beta cell dysfunction [29]. HOMA insulin secretion only shows a moderate correlation with first-phase insulin secretion and our findings may therefore represent an underestimation of early beta cell decompensation [18, 30]. We are unable to assess the relative importance of hepatic and peripheral insulin resistance in our two groups.

We used self-reported measures of physical activity and dietary patterns rather than objectively measured covariates. However, both these measures have previously been shown to be associated with the risk of type 2 diabetes and all-cause mortality in the Whitehall II cohort [31, 32]. Our study population reflects the mix of people employed in the UK civil service in the mid-1980s and consequently includes a low number of South Asian individuals and an over-representation of white-collar workers. This may somewhat limit the generalisability of our findings, although ethnicity was shown to be associated with incident diabetes in this cohort, with similar relative risks to those of other population-based studies [32]. Furthermore, we were unable to investigate whether fasting and 2 h glucose concentrations reflect different disease variants, due to the low number of incident diabetes in South Asian participants [15]. Finally, while we were able to adjust for a range of confounders, we cannot rule out residual confounding by unmeasured covariates such as birthweight and inflammatory markers.

In terms of public health recommendations, the earlier and faster increases in FPG found in our study suggest a shorter window for interventions to prevent type 2 diabetes in South Asians. The clinical implications of our findings are less certain, although some speculate that incretin-based treatments might be particularly suitable for South Asians with diabetes [8].

### Conclusions

Our description of ethnic differences in glucose, insulin, insulin sensitivity and insulin secretion before diabetes diagnosis supports the growing literature suggesting that there are important differences in the natural history of type 2 diabetes between South Asian and white individuals. Our findings support a much earlier role of beta cell dysfunction in South Asians, leading to rapidly increasing FPG trajectories and an increased risk of diabetes. Compared with white individuals, the earlier and faster increases in FPG among South Asians suggest that using the standard diabetes diagnosis cut-off will find higher risk people who have advanced deterioration of both insulin sensitivity and secretion. Our results support government recommendations to provide lifestyle intervention at an earlier age and at lower glucose values among South Asian populations.

## Abbreviations

2hPG: 2 h post-load plasma glucose
2hSI: 2 h post-load serum insulin
FPG: Fasting plasma glucose
FSI: Fasting serum insulin
HOMA2-B: HOMA of insulin secretion
HOMA2-S: HOMA of insulin sensitivity
ISI_0,120_: Gutt insulin sensitivity index
